# Molecular Characterization of *Cryptosporidium* Species in Children with Diarrhea in North West of Iran

**Published:** 2015

**Authors:** Behroz Mahdavi Poor, Jalil Rashedi, Mohammad Asgharzadeh, Esmaeel Fallah, Kareem Hatam-Nahavandi, Abdolhossein Dalimi

**Affiliations:** 1 *Department of Laboratory Science, Faculty of Paramedicine, Tabriz University of Medical Sciences, Tabriz, Iran. *; 2 *Department of Medical Parasitology, School of Medical Sciences, Tarbiat Modares University, Tehran, Iran. *; 3 *Biotechnology Research Center and Faculty of Paramedicine, Tabriz University of Medical Sciences, Tabriz, Iran. *; 4 *Department of Parasitology, Faculty of Medicine, Tabriz University of Medical Sciences, Tabriz, Iran.*; 5 *Department of Medical Parasitology and Mycology, School of Public Health, Tehran University of Medical Sciences, Tehran, Iran.*

**Keywords:** Child, * Cryptosporidium*, genotype, PCR, Iran

## Abstract

*Cryptosporidium* is one of the most common causes of childhood diarrhea in developing countries. The aim of this randomized pilot study was to detect and characterize infective species and determine the genotypes of *Cryptosporidium* parasites in pediatric patients suffering from diarrhea in North West of Iran. A total of 113 fecal samples were collected from diarrheic children hospitalized in Tabriz Pediatric Hospital. The amplification of small subunit ribosomal RNA gene was performed using a nested polymerase chain reaction protocol and its products were digested using two restriction enzymes for *Cryptosporidium* species and genotype differentiation. *Cryptosporidium *oocysts were found in 2 (1.76%) children with diarrhea and restriction pattern revealed the presence of *C.parvum *bovine genotype in both positive fecal samples. The findings indicate that *Cryptosporidium parvum *is responsible for cryptosporidiosis in children in the study region and probably zoonotic transmission is the predominant route of parasite transmission.

*Cryptosporidium* ssp. is an intracellular parasite that infects the epithelial margin of gastrointestinal and respiratory tracts in a wide range of hosts. The life cycle of the parasite is monoxenous and becomes completed within the gastrointestinal tract of a single host (1). It is responsible for considerable diarrheal disease affecting mostly children and immunocompromised individuals, especially in HIV positive patients (2-3).

Five species of * Cryptosporidium *including *C. *

*hominis* (previously known as the *C. parvum* human genotype), *C. parvum* (bovine genotype), *C. meleag-ridis*, *C. canis* and *C. felis* have been found to be responsible for most human infections. *C. hominis* is an anthroponotic genotype exclusively found in humans, while *C. parvum* (zoonotic genotype) is found in humans and wide range of domestic and wild animals (4).

Cryptosporidiosis occurs by consumption of ubiquitous oocysts which are transmitted indirectly via contaminated drinking and recreational water or directly via contact with infected humans or animals (1). The oocysts of parasites are environmentally highly resistant to common disinfectants and can survive in water for weeks (5). They are excreted in large numbers by infected humans and animals, and by means of discharging of waste, can reach surface water and consequently contaminate drinking water. The oocyst transmission via contaminated drinking water, recreational water and municipal water is well -proven, but probably drinking water has the most important role in the transmission of the infection, and proper treatment of drinking water leads to significant reduction in cases of disease(1,6).

None of common diagnostic laboratory techniques, such as acid fast staining, direct or indirect immunofluorescence microscopy allow species and genotype discrimination of the oocysts (1). The new tools of molecular genetics allow the characterization of *Cryptosporidium* at the species and genotype level, and also tracing different transmission ways of the parasite. The genetic techniques including PCR and restriction fragment length polymorphism (RFLP) or sequence analysis can help to determine genotypes of the parasite and the possible source and harmful effect to human health (6).

There is little information about the geographic distribution and prevalence of *Cryptosporidium* species and genotypes in the East Azerbaijan province, northwestern part of Iran. This randomized pilot study assesses the public health impact and medical issues of *Cryptosporidium* in children, as one of the most susceptible groups at risk for the infection. Also, PCR-RFLP method is used to characterize the parasite at species and genotype level to achieve a better understanding of the epidemiology and transmission of infection in the study area.

## Materials and methods

**Samples collection**

Fecal samples were collected from 113 children (age range: 3 months-12 years) with acute gastroenteritis hospitalized in Tabriz Pediatric Hospital, a referral center in the North West of Iran. The average age of children was 3 years old. Each sample was divided into two parts. One part of the specimen was concentrated by formalin- ether concentration method and the other part stored in 2.5% potassium dichromate solution and kept at 4 °C for further molecular analysis. The concentrated specimens were stained with acid- fast staining method and examined microscopically (7-8).

**DNA extraction**

Positive fecal samples and forty negative fecal samples which were randomly selected, were subjected to DNA extraction. About 200 mg of fecal materials that were stored in 2.5% potassium dichromate were rinsed five times with a solution of phosphate buffered saline (PBS, pH = 7.2) and centrifuged at 14000 g for 10 min at 4 °C to remove potassium dichromate completely. The pellets were subjected to ten freeze -thaw cycles (three min freeze in liquid nitrogen followed by three min at 65 °C). DNA was extracted by using the modified proteinase K (Fermentas, Lithuania), sodium dodecyl sulfate (SDS) (Merck, Germany) and cetyltrimethylammonium bromide (CTAB) (Merck, Germany) method (9). The extracted DNA pellets were dissolved in 20 μl of Tris-EDTA (TE) buffer and stored at -20 °C before application in PCR (2).

**PCR amplification and RFLP**

The amplification of small ribosomal subunit

 RNA (18S rRNA; SSU-rRNA) gene was performed using a nested PCR protocol, described previously (2, 10). Both positive (*Cryptosporidium* DNA) and negative (containing all PCR reagents without DNA template) controls were applied in each PCR round to validate the results. Depending on species and genotype in secondary PCR step, primer sets may amplify a range of 826- 864 bp fragments (6, 11).

For restriction fragment analysis, the secondary PCR products were digested by using SspΙ (Fermen-tas, Lithuania) for the differentiation of *Cryptospori-dium* species and VspI (Fermentas, Lithuania) for further differentiation between the two genotypes of *C.parvum*. Restriction enzymes were used under conditions recommended by the supplier. Digestion products were fractionated on a 2.0% agarose gel and visualized under ultraviolet (UV) light by ethidium bromide (0.5 μg/ml) staining and electrophoresis data were recorded using a UV transilluminator(8). Characterizations of the species and genotypes were performed according to the restriction patterns described previously (6).

## Results

The* Cryptosporidium *oocysts were found in 2 (1.7%) children with diarrhea by staining method. Both of the infected children were rural residents*. *By DNA amplification of the* Cryptosporidium* positive samples, we obtained products of the predicted size corresponding to about 830 bp in the nested-PCR analysis. Forty randomly selected negative samples that were tested with nested-PCR, did not show the expected product.

**Fig. 1:**
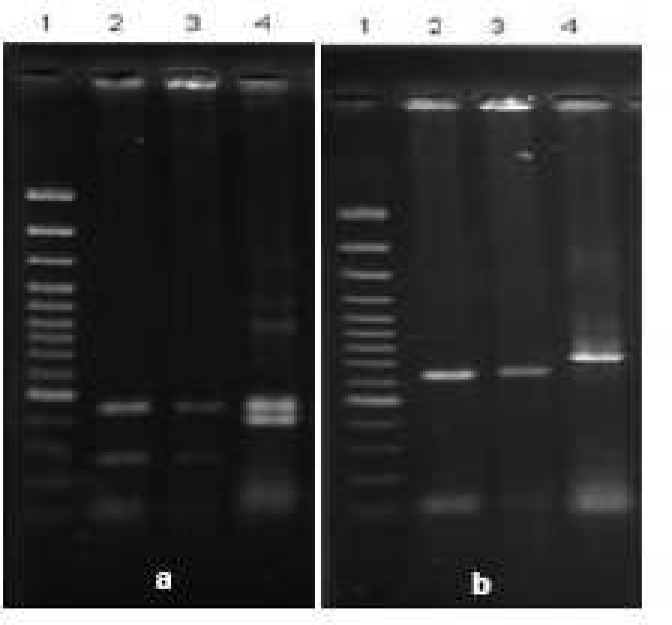
**a.** Digestion of secondary PCR product with* SspΙ*. Lane 1: 100 bp DNA ladder, lane 2 and 3: digestion product of *C.parvum* and lane 4: digestion product of *C.muris/andersoni* (cattle isolate). **b.** Digestion of secondary PCR product with* VspI*. Lane 1: 100 bp DNA ladder, lane 2 and 3: digestion product of *C.parvum* (bovin genotype) and lane 4:digestion product of *C.muris/andersoni* (cattle isolate).

The secondary PCR products were subjected to SspI restriction enzyme digestion for species differentiation and revealed the presence of *C.parvum *in both *Cryptosporidium* positive fecal samples as three bands of 449, 254 and 108 bp were visualized in gel electrophoresis (6), (Fig.1a).To distinguish human and bovine genotypes of *C. parvum*, the products of second PCR step were digested with VspI. *C. parvum *bovine genotype showed two visible bands of 628 and 104 bp (6) (Fig.1b).

## Discussion

The present pilot study attempted to detect* Cryptosporidium* and identify its species and the transmission patterns of infection. The findings indicate that *Cryptosporidium* is one of the causative agents of diarrhea in children and *C. parvum *(bovine genotype) is responsible for cryptosporidiosis in this group.

In this research, we amplified SSU-rRNA by the nested PCR technique. Because of the nested format of the PCR and multi -copy property of SSU- rRNA gene (five copies per sporozoite in *C.parvum* bovine genotype), this method is one of the most sensitive PCR protocols for *Cryptosporidium* oocysts detection in stool samples(6).

There are different reports on the prevalence of infection in diarrheic children from various regions of Iran. In the studies conducted by Keshavarz et al. (12) and Saneian et al. (13) in the central part of Iran, a low prevalence of about 2.5% and 4.6%, respectively, was detected. In contrast, the results of a study conducted by Mirzaei (14) showed high prevalence of about 35% in the southern part of Iran. This reflects geographic differences in the prevalence of *Cryptosporidium* in various part of Iran.

The disease in humans is mainly caused by two species of* C.parvum *and *C.hominis* (15). While the reservoir hosts for *C.parvum* are cattle, domestic livestock and humans, but the reservoir host for *C.hominis* is human. Distribution of two prevalent human infective species of parasites varies in different communities and countries. In the United Kingdom, *C.parvum* is responsible for most human cases (61.5%) (16) with a difference in seasonal pattern between two species. Moreover, in this country, *C.hominis* infection peak is in the Fall season whereas,* C.parvum* infection peak is in the late Spring season (17). Our study was carried out at the end of the Spring and findings are similar to the results of the studies in United Kingdom. In the United States, Australia, Kenya, Guatemala and Peru, most human cases are caused by *C.hominis *(18-19). Various reports indicate that *C.parvum* is more common than *C.hominis* in Iran and its neighboring countries (12, 20-23). In Turkey, the neighboring country of Northwest of Iran, among 707 elementary school children 4 (0.6%) cases were infected with *Cryptosporidium* that all isolates belonged to *C.parvum* bovine genotype (20). Our findings are similar to the results of other studies conducted in Iran, Saudi Arabia, Turkey and Kuwait.(12, 19-22). In the present study, the existence of *C.parvum* implies that the zoonotic transmission may be predominant in study region and neighboring areas.

Cattle are one the most important sources for the zoonotic cryptosporidiosis. Direct contact with infected calves or contamination of food or water by cattle manure can be one of the transmission routes of the zoonotic disease (4). A previous survey in Northwestern part of Iran showed that 10.5% of cattles had been infected with *C.parvum* and *C.andersoni*. Of the infected cattle, 36% of cases were related to* C.parvum* (2). The prevalence of this potentially zoonotic species in cattle and its presence in diarrheic rural children would strengthen the possibility of zoonotic transmission via direct contact with farm animals in this region.

In conclusion, the existence of the cryptosporidial infection in the children of the study area is an important warning for other more susceptible individuals. The presence of *C. parvum* may be associated with zoonotic transmission of the parasite. Therefore, cattle and other domestic animals should be considered as important sources of infection in the study area. However, further epidemiological studies are needed to assess accurately the risk of the cryptosporidial infection in other sensitive humans such as immunocompro-mised patients.
